# Subjective wellbeing at different spatial scales for individuals satisfied and dissatisfied with life

**DOI:** 10.7717/peerj.6502

**Published:** 2019-02-21

**Authors:** Ida Kubiszewski, Nabeeh Zakariyya, Diane Jarvis

**Affiliations:** 1 Crawford School of Public Policy, Australian National University, Canberra, Australia; 2 Research School of Economics, Australian National University, Canberra, Australia; 3 College of Business, Law & Governance, James Cook University of North Queensland, Townsville, QLD, Australia

**Keywords:** Wellbeing, Life satisfaction, Global progress, Spatial scales, Australia

## Abstract

Indicators that attempt to gauge wellbeing have been created and used at multiple spatial scales around the world. The most commonly used indicators are at the national level to enable international comparisons. When analyzing subjective life satisfaction (LS), an aspect of wellbeing, at multiple spatial scales in Australia, variables (drawn from environmental, social, and economic domains) that are significantly correlated to LS at smaller scales become less significant at larger sub-national scales. The reverse is seen for other variables, which become more significant at larger scales. Regression analysis over multiple scales on three groups (1) all individuals within the sample, (2) individuals with self-reported LS as dissatisfied (LS ≤ 5), and (3) individuals self-reporting LS as satisfied (LS > 5), show that variables critical for LS differ between subgroups of the sample as well as by spatial scale. Wellbeing measures need to be created at multiple scales appropriate to the purpose of the indicator. Concurrently, policies need to address the factors that are important to wellbeing at those respective scales, segments, and values of the population.

## Introduction

Over the past few decades, dozens of wellbeing indicators have been developed as a means of tracking societal progress, comparing countries, or developing policies (Smith et al., 2013). These indicators are frequently multi-faceted, reflecting the influence of environmental, social, and economic factors on wellbeing. Many of these indicators aggregate variables, either objective or subjective, to determine an overall wellbeing of a population. However, aggregating wellbeing indicators at different spatial scales may create measurement inconsistencies and, potentially, unreliable conclusions may be drawn.

Most indicators are created to measure average wellbeing at a national level, while a few focus on a single small community. There are advantages and disadvantages to using either scale. An indicator at the national scale allows for tracking of overall national progress and for cross-country comparisons. However, national level indicators aggregate a large number of individuals. This aggregation process may overlook critical information about portions of the population that have the dangerously low wellbeing and are most in need of community and government support (Andreasson, 2018). It also ignores the different value systems that distinct sub-groups of a community, especially minorities, may hold. Examples of indicators often used at the national scale include the sustainable development goals, human development index calculated by the United Nations, the genuine progress indicator, world values survey, happy planet index, and OECDs better life index (Kubiszewski et al., 2013; Costanza et al., 2014).

At the opposite end of the spatial scale, small “boutique” community indicators have grown in popularity around the world (Cox et al., 2010; Biedenweg et al., 2014). These indicators are developed and calculated for a specific community. This means that the indicator components are often customized to ensure the values of that community are represented. The benefit of calculating a wellbeing indicator at an individual and community scale is that it guarantees that those with extremely low wellbeing can be identified and provided with the support they need. However, because these measures usually focus on one specific community or portion of the population (Phillips-Howard et al., 2010; D’Acci, 2011; Vazi et al., 2013), they are often not transferable to other populations and locations. Such indicators prevent comparisons between regions and provide minimal information for regional level policies.

In addition to scale, the variables used to calculate a wellbeing indicator define how that indicator can be used and what aspect of societal progress it measures. The structure and composition of wellbeing indicators has varied significantly, and has included (1) adjusted economic measures, that go beyond GDP to incorporate aspects outside of the market economy, (2) subjective measures collected through surveys, and (3) weighted composite measures (Costanza et al., 2014).

In this paper, we analyze the relationship between categories 2 and 3, from the list above. We use life satisfaction (LS), one component of wellbeing that looks at individuals’ satisfaction with their own lives, as the subjective measure. Here, we consider objective variables as predictors, or correlates, of subjective LSs. However, we acknowledge that objective variables can also be measures, or proxies, of wellbeing itself, independently of subjective LS. Subjective LS may vary depending on cultural norms or individual perceptions (Dolan, Layard & Metcalfe, 2011; Graham, 2011; Diener, 2012). Also, the connection between an objective view of an individual’s circumstances and an individual’s own self-reported subjective LS may not be directly perceived due to a lack of information, distorted media portrayals, personality traits, individual’s limited information processing abilities, and cultural factors (Kahneman, 2011). This creates a situation where observation (objective variables) and perception (subjective variables) differ (Kubiszewski, Zakariyya & Costanza, 2018). However, perceptions have been found to be more an important determinants of behavior, or satisfaction with life, than measurable (“objective”) variables (Schneider, 1975; Cummins, 2000).

One example where perceptions and observations can become disconnected is around crime. Polices reducing crime levels may be effective, but the perception of crime may remain high. This may occur as a consequence of a personal experience of crime, lack of satisfaction with community cohesion, concern with the opportunities for the next generation, anxiety over the state of the economy, or simply overemphasis of crime in the media (Tyler & Boeckmann, 1997; Duffy et al., 2008; King & Maruna, 2009; Ambrey, Fleming & Manning, 2014). Nevertheless, the subjective LS of an individual is a major contribution to a person’s overall wellbeing, regardless of the causes influencing the individuals perception of their LS (Diener, 2009).

However, the majority of wellbeing indicators focus on the use of objective variables. Objective variables are generally easier to measure and to directly target through policy interventions at societal scale. They can also target aspects that contribute to wellbeing but may not be perceived by individuals. For example, ecosystem services, such as water purification or carbon sequestration, are not directly perceived by individuals but are crucial to human wellbeing (Costanza et al., 2017). Inequality has also been shown to have a significant impact on wellbeing but is often not directly perceived, or if it is, only at certain scales (Wilkinson & Pickett, 2006, 2009).

How a wellbeing indicator is structured, and which variables are used, needs to reflect the values, goals, and cultural priorities of the individuals within that society. Once those variables are established, and their contribution to LS and wellbeing verified, aggregated measures can be used to monitor regional or national progress. These indicators can then influence people’s daily lives by shaping policy. Understanding how policies, local and sub-national, are perceived and at which spatial scales they are perceived, will improve understanding of the impact of those policies (Diener & Suh, 1997; Cash et al., 2006). Furthermore, data regarding LS levels, and the factors contributing to this, are likely to contain non-stationary and geostatistical properties, changing over the course of an individual’s life, requires that policies be adaptive and be re-evaluated at regular intervals. It is therefore critical that the relationship between objective and subjective variables is understood, and that this understanding meets the needs of a diverse populations rather than prioritizing the elite (Bache, Reardon & Anand, 2016; Cairney et al., 2017).

Although extensive research has been published around scaling based on biophysical characteristics, little has focused around the relationship between wellbeing and spatial scale (Costanza & Maxwell, 1994; Cash et al., 2006; Hoffmann et al., 2015; Ettema & Schekkerman, 2016). As far as we are aware, there are no previous studies that have analyzed the impacts of aggregation on wellbeing indicators. We seek to address this research gap by systematically analyzing the impact of spatial scales on self-reported LS. Specifically, we examine how the relationship between overall subjective LS and various objective variables changes as individual data is aggregated over five different spatial scales within Australia. Furthermore, we assess not only the population as a whole, but also those that indicate a low level of LS, separately from those that indicate a high level of LS, finding important differences between these sub-populations. We map these results and the distribution of our objective variables across Australia.

## Methods

We estimate the relationship between subjective wellbeing, as measured by self reported LS, and aggregated objective variables representing natural, social, human, and built capital, over different spatial scales.

### Life satisfaction data

For this study, we aggregate overall individual level LS scores derived from waves 1–16 (collected in 2001–2016) of the Household, Income and Labour Dynamics in Australia (HILDA) Survey^1^1This paper uses unit record data from the Household, Income and Labour Dynamics in Australia (HILDA) Survey. The HILDA Project was initiated and is funded by the Australian Government Department of Social Services (DSS), and is managed by the Melbourne Institute of Applied Economic and Social Research (Melbourne Institute). The findings and views reported in this paper, however, are those of the author and should not be attributed to either DSS or the Melbourne Institute.. LS is taken from responses to the question, “All things considered, how satisfied are you with your life?” Responses are given on an 11-point Likert scale where 0 means totally dissatisfied and 10 stands for totally satisfied.

We acknowledge that calculating the mean of Likert items can be problematic, especially not knowing whether increments in scale correspond to equal increments in the underlying latent variable. Treating LS as ordinal vs interpersonally cardinally comparable is a contentious issue in literature. Justifications for cardinality shows that treating LS data as cardinal yields similar results to treating it as ordinal, and both assumptions are compatible with LS scores (Ferrer-i-Carbonell & Frijters, 2004; Blanchflower & Oswald, 2011; Kristoffersen, 2017). Further, Kristoffersen shows that LS scores are equidistant (Kristoffersen, 2017). The purpose of this paper does not require us to take a strong standpoint in this debate. Rather, our aim is to examine the consequences of aggregation commonly used in subjective wellbeing studies.

### Objective variables

The objective variables used in this study include eight related to human capital, four to social capital, and three to built capital (all based on data from the HILDA Survey discussed below). One natural capital proxy variable (normalized difference vegetation index (NDVI)) was included (discussed below) as no natural capital variables are collected as part of the HILDA Survey. Natural capital has been shown to have a significant impact on LS (Ambrey & Fleming, 2014; Tsurumi & Managi, 2015; Fleming, Manning & Ambrey, 2016; Larson, Jennings & Cloutier, 2016). However, many LS and wellbeing studies omit reference to the environmental domain entirely (Helliwell, Layard & Sachs, 2018). The choice of variables for inclusion within this study were identified based on outcomes from previously published literature, including a similar study (utilizing HILDA data) performed at the individual scale (Kubiszewski, Zakariyya & Costanza, 2018).

The objective variables were aggregated for individuals living within a given geographic area. We aggregated continuous variables by calculating the mean value per given area. For example, the mean household disposable income for a given area was calculated. Categorical variables were aggregated by obtaining the proportion of individuals of a specific category out of the total individuals within each respective area; for example, the proportion of men, the proportion of university graduates, and the proportion of those with a long-term health condition within each area.

#### Natural capital data—NDVI

We use the NDVI as a proxy for natural capital. NDVI measures the amount of live green vegetation present. The source of the NDVI data is the Australian Government Bureau of Meteorology (http://www.bom.gov.au/jsp/awap/ndvi/archive.jsp?colour=colour&map=ndviave&period=month&area=nat), derived from satellites.

Normalized difference vegetation index is an index measuring the difference between visible light absorbed and infrared radiation reflected by vegetation. This measure changes due to vegetation density and greenness. The index value lies between −1 and +1. Higher values are associated with greater density and greenness, decreasing as vegetation comes under water stress, becomes diseased, or dies. Bare soil and snow values are close to zero, while water bodies have negative values.

For this analysis, we use NDVI values from January of each year of the HILDA Survey to ensure the data reflects variations year to year. January was selected as being in the middle of the growing season, thus being likely to reflect the period of maximum greenness. NDVI is primarily used as a means of comparison from year to year and between scales. In this study, it is not used for its absolute value.

Each years’ January data (in the form of a spatial file using a 0.05 × 0.05 degree grid) was intersected with files containing the boundaries of the statistical geographic scales used in this paper. The average NDVI score per geographic region, weighted by spatial area, were calculated using the proportion of each region’s area represented by different NDVI scores. These scores per region were calculated at each scale in turn, providing the data for inclusion within the regression described above.

We considered the use of other measures of natural capital in our model, including ecosystem services values, land cover, and a weather index, but found that none of these were significant, nor ideally suited to the core question examined in this paper. The fine-grained spatial detail of the NDVI dataset is ideal for investigating the impact of aggregating data across different scales. The ecosystem services values and land cover datasets considered were less finely grained. Also, when using the value of ecosystem services, especially certain land covers, the greater value is often derived from those services that are not perceived (Costanza et al., 2011). For example, wetlands are often perceived as useless land while providing significant contributions to humans living across a wide geographic area rather than just to those living within the immediate vicinity. The aggregation of weather measures across different spatial scales presents their own challenges^2^2For example, when estimating the areal rainfall across a large geographic area the selection of the appropriate rain stations data to use, and how this should be aggregated, forms an entire body of literature of its own.. Although NDVI is also not directly perceived, it does provide a more direct proxy for natural capital as it shows location of all nature and its degradation (Bai et al., 2008).

### Separating “satisfied” and “unsatisfied” individuals

We also divided our total sample into two subsamples. These subsamples separately considered individuals who responded as having a LS score between completely dissatisfied (0) to neutral (5) and individuals who responded as having a LS score between just above neutral (6) and completely satisfied (10). For brevity, we refer to the full sample as “All,” sample 2 as “Satisfied,” and sample 3 as “Unsatisfied.” Using LS subsamples as a means of understanding different portions of the population has been previously used in literature (Andreasson, 2018). In this paper, we use two sub-samples as our total sample is not large enough to split further.

Besides our current approach of separating “satisfied” and “unsatisfied” at a response of 5 and below and above 5, we also attempted to separate the total sample through other methods. In this respect, we attempted creating separation points using the median of LS and LS quartiles. Both of these separation points showed insufficient variation between the subsamples and the sample size were too small to make an informed analysis. Our two current subsamples provide a meaningful split in the population in determining what are the different contributions to the LS for each of the subsamples and why. Most importantly, it gives us additional information on the portion of the population that is most at risk.

### Estimation strategy

We examine the relationship between average LS and objective variables using the following empirical model:

(1) $${\rm{Average\; L}}{{\rm{S}}_{i,t}} = {\beta _0} + {\beta _1}{X_{i,t}} + {\lambda _t} + {u_{i,t}}$$

where Average LS_*i*,*t*_ is average LS of individuals in area *i* in year *t*, *X_i,t_* are the objective variables for area *i* in year *t*, λ_*t*_ is the year fixed effect, and *u_i,t_* is the area level idiosyncratic error.

Our regression model uses pooled ordinary least squares controlling for time fixed effects and using cluster-robust standard errors. Our choice of estimation method is based on a number of factors. The Hausman test indicates the possibility of unobserved characteristics being correlated with the objective variables included in the model, for all samples, (at 1% significance level for SA1–SA4 and at 5% level for state) thus the use of random effects estimation would be inappropriate.

We also ruled out extremely large time invariant area level fixed effects. Our unit of estimation is not the individual but an aggregate of individuals within a given statistical area. It is important to note that the composition of individuals being aggregated within a given area changes across the years. Table 1 shows the percentage of individuals who have moved to a new area in each year in our “All” sample. As expected, there is greater movement, or relocation, at smaller spatial scales compared to larger scales. In addition, 5,477 individuals were added to the HILDA Survey in wave 11 (2011).

**Table 1:** Number of areas and individuals within an area at the various scales.

	SA1	SA2	SA3	SA4	State
	Sample “All”				
Average number of areas 2001–2016	4,581.6	1,509.4	317.1	87	8
Standard deviation between years	(1,570.0)	(301.2)	(12.13)	(0)	(0)
Average number of individuals within an area	3.2	9.75	46.41	169.10	1,839.40
Standard deviation between areas (averaged across years)	(3.99)	(10.21)	(32.44)	(88.95)	(1,603.40)
	Sample “Unsatisfied” (LS ≤ 5)				
Average number of areas 2001–2016	773.2	570.3	257.8	85.50	8
Standard deviation between years	(120.2)	(76.43)	(9.270)	(1.155)	(0)
Average number of individuals within an area	1.289	1.748	3.867	11.66	124.6
Standard deviation between areas (averaged across years)	(0.706)	(1.228)	(2.947)	(7.696)	(110.5)
	Sample “Satisfied” (LS > 5)				
Average number of areas 2001–2016	4,408.9	1,487.8	316.7	87	8
Standard deviation between years	(1,522.5)	(304.3)	(12.27)	(0)	(0)
Average number of individuals within an area	3.112	9.220	43.32	157.7	1,714.8
Standard deviation between areas (averaged across years)	(3.796)	(9.592)	(30.28)	(82.71)	(1495.1)

**Note:**

This table shows the average number of areas, average individuals per area, and the standard deviation for both at all five scales. The three samples analyzed are (1) all individuals within the 16 waves that responded to the life satisfaction question, (2) individuals that replied that their life satisfaction was equal or less than five, and (3) individuals that replied that their life satisfaction was greater than five.

We conducted Fisher-type Augmented Dickey Fuller tests under various assumptions on each of the SA levels to ensure that aggregate LS within areas was stationary. The tests strongly reject the null hypothesis that all the panels contain unit roots (*p* < 0.01). Although the LS of individuals may not be stationary over the course of a lifetime, we do not use individual LS but aggregate LS in this paper. Aggregated data is prone to be more stationary as individuals within the aggregated data vary in age and may migrate between areas. Regardless, we acknowledge that unobserved area level fixed effects could be present. Nevertheless, our main interest lies in variation between areas rather than within areas over time. Most importantly, the aim of this paper requires us to examine how the preciseness of the estimates changes as we move up the spatial scale. We do not make strong causal or correlational inferences on the relationships.

### Aggregation and empirical model

The three samples analyzed include, (1) all individuals within the 16 waves that responded to the LS question, (2) individuals that replied that their LS was equal or less than five, and (3) individuals that replied that their LS was greater than five. The latter two samples enable us to examine how information relevant to individuals, or small groups, particularly for those at opposing ends of the LS Likert scale, is lost as we aggregate.

For each of our samples, we aggregate individual level data to five spatial scales. The spatial scales used in this paper are based on the Australian Statistical Geography Standard (ASGS) hierarchical scales to allow for future comparison. Listed in ascending order, they include Statistical Area Level 1 (SA1), Statistical Area Level 2 (SA2), Statistical Area Level 3 (SA3), Statistical Area Level 4 (SA4), and State and Territory (State).

The Australian Bureau of Statistics designed the SA geographic structure specifically for the release of statistical information (http://www.abs.gov.au/websitedbs/D3310114.nsf/home/geography). Their sizes are based on population, not area, and each SA level aggregate up to the next SA level. SA1s have populations of approximately 400 (between 200 and 800) people and aim to separate differing geographical characteristics. SA2s have average populations of about 10,000 (between 3,000 and 25,000) people and were designed to represent communities that interact economically and socially. SA3s have between 30,000 and 130,000 people and were designed to include an entire region that contains a small town or suburb. SA4s have between 100,000 and 300,000 people and were setup to reflect labor markets within regions. States and territories boundaries are defined by the federal government.

Table 2 presents the total number of areas at each scale and the average number of individuals within a given area at each scale. For all years, our sample includes 87 SA4 areas and eight states/territories. The number of SA1, SA2, and SA3 areas vary from year to year, depending on location of respondents. On average there are 4,581 SA1 areas, 1,509 SA2 areas, and 317 SA3 areas. The SA1s, on average, include 3 (±3.99 SD) individuals, SA2s include 9 (±10.21 SD) individuals, and SA3s include 46 (±32.44 SD). The variation in the number of individuals within an area increases as we move up the spatial scale and consider larger areas. These deviations impact aggregation from individual level data within an area. There is greater dispersion in individual responses as the number of individuals within an area increases along with spatial scale.

**Table 2:** Percentage of individuals who have moved to a new area in each year from the previous year.

	SA1	SA2	SA3	SA4	State
2002	10	8	6	4	1
2003	16	13	9	6	1
2004	16	12	8	6	1
2005	16	12	9	6	2
2006	15	12	8	6	2
2007	16	12	9	7	2
2008	15	12	8	6	2
2009	15	12	8	6	1
2010	16	12	9	6	1
2011	11	9	6	4	1
2012	15	12	9	6	2
2013	16	12	9	6	2
2014	16	12	9	6	2
2015	16	12	9	6	2
2016	16	13	9	6	2

**Note:**

This is for the “All” sample.

## Results

### Descriptive statistics

To understand the relationships between objective variables and average LS at different spatial scales, it is important to examine how the aggregation of these objective variables differs across the spatial scales. Table 3 presents the mean and standard deviation (reported in parentheses) of the average LS for the three samples.

**Table 3:** Average life satisfaction and average variation in life satisfaction within areas at each scale.

	SA1	SA2	SA3	SA4	State
	Sample “All”				
Average life satisfaction within an area	7.808	7.845	7.916	7.943	7.923
(SD of LS between areas)	(1.235)	(0.909)	(0.428)	(0.216)	(0.0873)
Standard deviation of life satisfaction within an area	0.694	1.109	1.405	1.467	1.470
	(0.808)	(0.734)	(0.391)	(0.184)	(0.119)
	Sample “Unsatisfied” (LS ≤ 5)				
Average life satisfaction within an area	4.243	4.248	4.239	4.231	4.214
(SD of LS between areas)	(1.121)	(1.025)	(0.786)	(0.487)	(0.245)
Standard deviation of life satisfaction within an area	0.172	0.358	0.747	1.061	1.190
	(0.519)	(0.684)	(0.736)	(0.515)	(0.310)
	Sample “Satisfied” (LS > 5)				
Average life satisfaction within an area	8.094	8.125	8.182	8.205	8.181
(SD of LS between areas)	(0.891)	(0.663)	(0.327)	(0.176)	(0.0764)
Standard deviation of life satisfaction within an area	0.539	0.843	1.056	1.095	1.095
	(0.586)	(0.498)	(0.222)	(0.0828)	(0.0479)

**Note:**

This table shows the average life satisfaction for the three samples and the standard deviation within an area. The numbers beneath the average LS (reported in parentheses) are the standard deviations between the different statistical areas.

As we aggregate over larger areas, the population within an area becomes more diverse, but the diversity between the areas decreases. At SA1, the average LS is 7.81 with a standard deviation of ±1.24 between the SA1 scales, compared to the standard deviation of LS within an SA1 area, which is ±0.69. This implies that at the SA1 level, individual LS scores between areas are more dispersed than the aggregated scores within an area. This is reversed as the scale increased. The standard deviation of average LS decreases between areas as the spatial scale increases in size, being largest at SA1 and smallest at the state level. The states and territories have fairly similar average LS scores to each other (average of 7.92 ± 0.087). This also indicates that key information about dispersion of individual LS scores is lost as we aggregate across larger spatial scales (and more individuals). This is an important finding that is reflected in our regression results.

**Figure 1:**
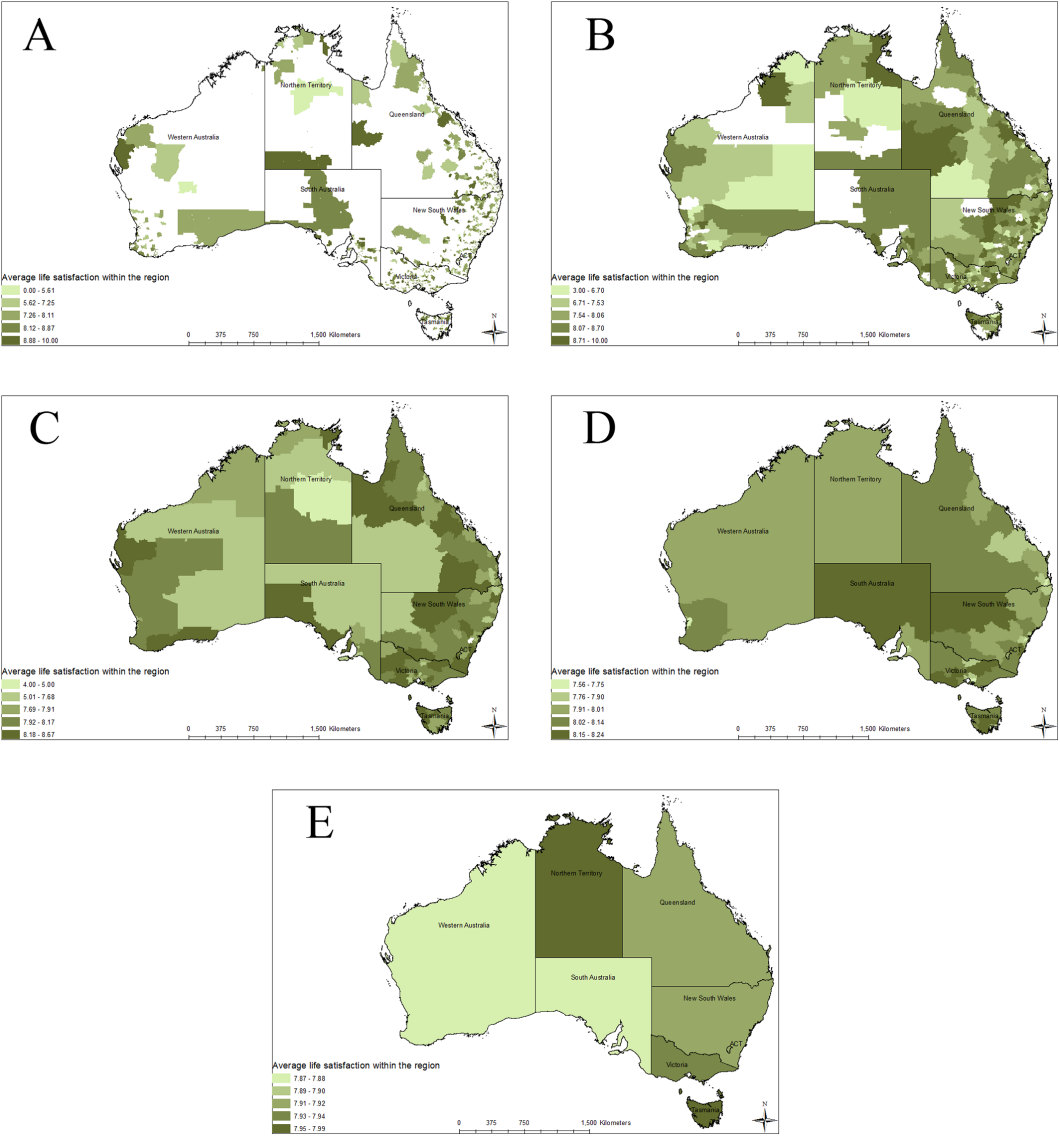
Average life satisfaction with the region at various spatial scales. This figure shows the spatial scale distribution of life satisfaction within Australia over five different spatial scales. It also shows how aggregation to larger scales loses information about individuals below a certain average life satisfactions. The five different spatial scales are: (A) SA1, (B) SA2, (C) SA3, (D) SA4, and (E) state and territory.

This can be seen when the average LS is mapped across Australia at the five different spatial scales (Figs. 1A–1E). The differences between the different areas are significantly decreased as the aggregation areas grow. Populations seen in Fig. 1A (SA1) that report average LS between 0 and 5.61 no longer show up in Fig. 1E (state/territory scale).

Average LS for the “Unsatisfied” sample is around 4.2 and 8.1 for the “Satisfied” sample, at all spatial scales. The “Satisfied” sample has greater standard deviation in individual LS within areas compared to the “Unsatisfied” sample at all scales except state. In contrast, the “Unsatisfied” sample as greater standard deviation between areas compared to the “Satisfied” sample.

Table S1 presents the descriptive statistics for the objective variables used in this paper. Similar to the standard deviation of average LS, the standard deviation of the objective variables between the areas decreases as the spatial scale increase. In some cases, large differences exist in the averages between the two samples. For example, the average household disposable income for the “Unsatisfied” sample is around $60,000 (±$59,000) at the SA1 level, while for the “Satisfied” sample it is around $80,000 (±$75,000). While at the state/territory scale, the average household disposable income for the “Unstatisfied” sample is about $63,000 (±$27,500) and $84,000 (±$23,000) for the “Satisfied” sample. The “Satisfied” sample is fairly similar to the full sample, which is expected as the “Satisfied” sample comprises 94% of the “All” sample.

Other variables seem to have major differences between the “Unsatisfied” and “Satisfied” samples. For example, at SA1, in the “Unsatisfied” sample the average portion of individuals that don’t exercise at all is about 0.2% and in the “Satisfied” sample, this is around 0.08%.These averages are similar at all scales. We also see that the proportion if individuals with a bachelor’s degree or higher qualification is great in the “Satisfied” sample as is the proportion of individual’s in a relationship, and the number of hours worked per week. The objective variables that seem to be smaller at all scales in the “Satisfied” sample include the proportion of individuals that are unemployed, divorced, engage in no physical activity at all (as discussed above), and having a long-term health condition.

### Regression results

The objective variables chosen for this regression were based on those that had a highly significant correlation to subjective LS at the individual level (*p* ≤ 0.05) based on previous research, and at SA1 scales. The number of observations that are stated in Table 4 indicate the number of SAs over the 16 years which contain respondents to the overall LS question in the HILDA Survey. There are 62,499 SA1s that have at least one person that responded to the LS question in the HILDA Survey. There are 9,484 SA1s over the 16 years that have at least one person with a LS equal or less than 5 and 60,141 with at least one person with a LS of greater than 5.

**Table 4:** Regressions correlating average subjective life satisfaction (dependent variable) and a set of objective variables (independent variables) over five scales.

	Sample “All” (1)					Sample “Unsatisfied” (LS ≤ 5) (2)					Sample “Satisfied” (LS >5) (3)				
	SA 1	SA 2	SA 3	SA 4	State	SA 1	SA 2	SA 3	SA 4	State	SA 1	SA 2	SA 3	SA 4	State
Age (last birthday on June 30, 2016)	−0.0349***	−0.00788*	0.00465	0.0471	−0.0726	−0.0159***	−0.00155	0.0374***	0.0216	−0.00902	−0.0235***	−0.0191***	−0.0155*	0.0414*	−0.112
Age squared	0.000462***	0.000203***	0.0000991	−0.00026	0.000633	0.000184***	0.0000307	−0.00038***	−0.000217*	0.000140	0.000321***	0.000298***	0.000280***	−0.00026	0.00110
Male (prop. of individuals)	−0.121***	−0.0937*	−0.136	0.368*	−0.0941	−0.0946***	−0.103***	−0.0925*	−0.00639	0.287	−0.0901***	−0.0938**	−0.0642	0.256	−0.376
Australia born (prop. of individuals)	0.107***	0.0846*	0.411***	0.540***	0.130	−0.0855*	−0.0762	−0.0218	0.110	0.0539	0.0868***	0.0422	0.359***	0.523***	0.146
Immigrant from English speaking country (prop. of individuals)	0.129***	0.199***	0.400***	0.0860	0.175	−0.111*	−0.0841	−0.0941	0.163	−0.0702	0.102***	0.0586	0.333***	0.186	0.191
Indigenous (Aboriginal/Torres strait) (prop. of individuals)	0.266***	0.113	0.659***	0.477**	−0.622	0.124	0.132	0.0283	−0.433	0.313	0.296***	0.230***	0.588***	0.309	−1.243*
Speaks English well (prop. of individuals)	0.458***	0.429***	0.0141	−0.0204	−2.51***	−0.0696	−0.0393	0.139	−0.446**	−0.965*	0.320***	0.368***	−0.106	−0.277	−2.63***
Unemployed (prop. of individuals)	−0.487***	−0.340***	−0.366	0.0176	−0.246	−0.00800	−0.00606	−0.0802	0.187	−0.0307	−0.241***	−0.130*	−0.200	0.115	0.177
Homeowner (prop. of individuals)	0.173***	0.149***	0.0442	−0.0421	0.276	0.0137	0.0280	−0.0503	−0.0123	0.0128	0.0853***	0.0835***	0.0359	−0.0166	0.0414
Household disposable income	0.191***	0.190***	0.113**	0.131***	−0.0807	0.0778***	0.0411	0.0683	0.0178	0.0385	0.0778***	0.0833***	0.111***	0.118***	−0.0751
Below poverty line (prop. of individuals)	0.0313	−0.0122	0.109	0.0895	1.042**	0.0844*	0.0809	0.0989	−0.0402	−0.0544	0.00582	0.0192	0.258***	0.104	0.826**
Bachelor’s or higher qualification (prop. of individuals)	−0.174***	−0.216***	−0.297***	−0.22***	−0.571	−0.126***	−0.172***	−0.139**	−0.138	0.0303	−0.104***	−0.140***	−0.245***	−0.151**	−0.624**
Hours worked at all jobs (per week)	−0.00576***	−0.00539***	−0.009***	−0.00338	0.0121	0.00431***	0.00363***	−0.000868	−0.00122	0.00220	−0.00589***	−0.00446***	−0.00737***	−0.00409	0.00709
Hours spent doing volunteer/charity work (per week)	0.0124***	0.0163***	0.0276***	0.00305	−0.0395	0.00257	0.000813	−0.00469	0.000489	0.0223	0.00760***	0.0136***	0.0242***	0.00501	−0.0442
Have 1 child (prop. of individuals)	−0.0670***	−0.0915**	−0.0387	0.0710	−0.337	−0.00250	0.0355	−0.00459	0.100	0.295	−0.0592***	−0.0296	0.0538	0.112	−0.335
Have 2 or more children (prop. of individuals)	−0.177***	−0.169***	−0.375***	−0.0591	0.347	−0.0253	−0.00317	−0.0733	−0.275*	0.175	−0.125***	−0.103***	−0.262***	0.0233	0.104
In a relationship (prop. of individuals)	0.346***	0.220***	0.358***	0.264*	0.484	0.113***	0.0951**	0.0134	0.166	0.00887	0.177***	0.140***	0.141*	0.329***	0.580
Separated or divorced (prop. of individuals)	−0.292***	−0.441***	−0.129	−0.440	−1.327	−0.111**	−0.119**	−0.191**	0.0975	0.151	−0.0415*	−0.0544	−0.0958	−0.245	−1.600*
Engages in no physical activity at all (prop. of individuals)	−0.278***	−0.258***	−0.0542	−0.0679	0.229	−0.204***	−0.194***	−0.112	0.0581	0.389	−0.0428*	−0.00455	−0.0749	−0.0496	0.188
Long-term health condition (prop. of individuals)	−0.681***	−0.562***	−0.649***	−0.38***	0.355	−0.137***	−0.131***	−0.180***	−0.250**	−0.223	−0.358***	−0.291***	−0.301***	−0.234**	0.294
Weighted average NDVI	0.153***	0.273***	0.0658	−0.0538	0.261**	0.185	0.0745	0.0552	0.148	−0.0101	0.107***	0.271***	0.0827*	−0.0457	0.223**
Any other adults present during survey (prop. of individuals)	−0.143***	−0.114***	−0.110*	−0.0735	0.130	0.00445	−0.0124	−0.0515	−0.148*	−0.140	−0.0848***	−0.0586***	−0.0719*	−0.0488	0.227*
Life satisfaction (sd at state level)	−0.258***	−0.301***	−0.438***	−0.49***	−0.50***	−0.435***	−0.464***	−0.563***	−0.681***	−0.677***	0.0507***	0.0851***	−0.0203	0.138	0.252
Constant	6.700***	6.145***	7.135***	5.463***	12.80***	3.969***	4.175***	3.293***	4.716***	5.301*	7.705***	7.330***	7.347***	5.324***	13.05***
Observations	62,449	22,745	5,022	1,392	128	9,484	7,518	3,779	1,351	128	60,141	22,401	5,018	1,392	128
Adjusted R-squared	0.156	0.174	0.338	0.516	0.701	0.087	0.145	0.303	0.543	0.794	0.082	0.082	0.199	0.415	0.715

Legend			
	Positive	Negative	
*p < 0.05			
**p < 0.01			
***p < 0.001			

**Note:**

The three samples analyzed are (1) all individuals within the 16 waves that responded to the life satisfaction question, (2) individuals that replied that their life satisfaction was equal or less than five, and (3) individuals that replied that their life satisfaction was greater than five.

The model goodness of fit as measured by the adjusted *R*^2^ increased with each increase in spatial scale (from 0.156 at SA1, through to 0.686 at state level for the full sample) (Table 4). This increase is to be expected as more information is being included in the model, increasing model fitness (Costanza & Maxwell, 1994). Moreover, as seen from Table 2, there is little deviation in average LS at the state level. Thus, the total sum of squares residuals is lower compared to smaller scales.

As the resolution of the scale decreases (aggregation area becomes larger), so does the uncertainty of our model as the estimated coefficients lose precision. This is seen in the higher robust standard errors relative to the coefficients. The increase in uncertainty is due to a larger number of people being aggregated in larger areas, overlooking individuals on the extremes. Further as evident from the standard deviations of the variables between areas (Table 3), there is little disparity between area level aggregates at higher scales.

#### Regression with all individuals, sample “All”

In the “All” regression, we find that most of the variables are highly significant at the SA1 level, except for the variable showing the proportion of individuals under the poverty line (*p* = 0.225). As the scale gets larger, estimates for more variables lose their precision and become statistically insignificant.

The results also highlight a few variables that remain significant across the spatial scales. The variables that are significant at all scales except state include: proportion of individuals that were born in Australia (positive) (*p* = 0.04 for SA2 and *p* < 0.001 for all other SAs), household disposable income (positive) (*p* = 0.004 for SA3 and *p* < 0.001 for all other SAs), educated to at least bachelor’s degree level (negative) (*p* < 0.001 for all SAs), being in a relationship (positive) (*p* = 0.004 for SA3 and *p* < 0.001 for all other SAs), and suffering a long-term health condition (negative) (*p* < 0.001 for all SAs). Those variables that are significant at scales SA1–SA3 include: proportion of individuals that immigrated from an English-speaking country (positive), hours worked (negative), hours spent doing volunteering or charity work (positive), and having two or more children (negative), all with *p* < 0.001. However, there are some variables that do not become significant until the state level, and others that change signs at the state level. The environment (as measured by NDVI) is highly significant at the smaller scales of SA1 and SA2 (*p* < 0.001).

The coefficient for the average standard deviation of LS within an area increases in magnitude and remains statistically significant (*p* < 0.001) as the spatial scale increases. The variation in individual LS within areas is higher at larger spatial scales. This has a significant effect in reducing the area level aggregated LS score.

#### Regression with individuals of life satisfaction ≤5, sample “Unsatisfied”

The loss of information from aggregation to larger scales is more evident when the full sample is divided into subsamples. The first subsample, “Unsatisfied” includes the number of individuals that have a LS score equal or less than 5 (approximately 6% of the individuals of the full sample).

Compared to the full sample, fewer variables were highly significant (*p* < 0.001) at each spatial scale, including SA1. There are also variables that, although remained statistically significant, changed signs from the full sample at SA1 level, including proportion of individuals that were born in Australia (now negative) (*p* = 0.019), immigrants from English speaking countries (now negative) (*p* = 0.032), and the number of hours worked per week (now positive) (*p* < 0.001).

For this sample, weighted average NDVI and proportion of individuals below poverty line are not statistically significant at any scale. Mean household disposable income is only significant at SA1 (*p* < 0.001). Another important difference is that time fixed effects are not statistically significant for this sample.

#### Regression with individuals of life satisfaction >5, sample “Satisfied”

The regression that includes those individuals with a LS score of greater than five, sample “Satisfied,” contains approximately 94% of all individuals in the sample and is similar to the “All” regression.

However, there are some important differences between regression “All” and regression “Satisfied.” NDVI is significant at all spatial scales, except at SA4 (*p* < 0.05). The proportion of individuals that identify as Indigenous is highly significant at SA1, SA2, and the state level (*p* < 0.001); however, it has a positive relationship at the first two scales, but a negative relationship at the state level. Another difference is that the proportion of individuals that do not engage in any physical activity only has a significant correlation at SA1 (*p* = 0.022).

## Discussion

One reason that consensus has not been developed around an overall wellbeing indicator stems from a disagreement around which variables contribute to wellbeing. This uncertainty may have evolved when assessing the contributors to LS and wellbeing at different scales and of different populations. Table 4 shows that different variables are significant depending on the scale. If a policymaker is trying to determine the impact that the proportion of individuals in poverty, or with a university degree, or with a long-term health condition, has on the LS within a community, the answer will depend on the scale adopted for the analysis. As this is true with many variables analyzed, developing consensus around a single indicator suitable for all scales may be impossible.

Many variables that are significant to individuals or at small spatial scales become statistically insignificant as the aggregation area grows. Variables such as unemployment, engages in physical activity, divorced, having children, and many others are highly significant and have the largest marginal effects at SA1 level (*p* < 0.001), are not significant at the higher scales of aggregation.

On the other hand, other variables only become significant at the state level. When looking at the “All” sample, the only variables significant at the state level are proportion of individuals that speak English well (negative coefficient) (*p* < 0.001), proportion of individuals that are below the poverty line (positive coefficient) (*p* < 0.005), NVDI (positive coefficient), (*p* = 0.028) and the standard deviation of LS (negative coefficient) (*p* < 0.001).

Variables, such as proportion of individuals that speak English well, change signs as the scale increases. In the sample “All,” the variable ‘proportion of individuals that speak English well’ is significant with a positive coefficient in SA1 and SA2 (*p* < 0.001), but becomes insignificant in SA3 and SA4. Interestingly, it becomes significant again at the state scale, but with a negative coefficient. At smaller scales, immigrants have been shown to have a more difficult time integrating into their communities and accessing basic needs like health care (Ku & Matani, 2001; Pitkin Derose et al., 2009). However, at a large scale, the diversity that immigrants bring to society outweighs the difficulties they may have (Borjas, 1990).

The variable “proportion of individuals that are below the poverty line,” is not significant at any level except state. One explanation is that individuals perceive poverty at only certain scales, as they do inequality within society (Subramanian & Kawachi, 2004; Wilkinson & Pickett, 2006; Boyce, Brown & Moore, 2010). Wilkinson (1997) argues that although the impacts of income inequality exists at small spatial scales, it is perceived at the larger societal scales. However, why this variable is positive is not clear and will require further study.

The natural capital variable, NDVI, is significant with a positive coefficient at SA1, SA2, and state level, but not at SA3 and SA4. Individuals utilize nature in different ways. Local parks, gardens, and trees liven up an urban area at the community level. They can be seen and enjoyed every day (Brereton, Clinch & Ferreira, 2008). At the state level, nature can be enjoyed in the form of national parks through hiking, camping, and other recreational activities (Maller et al., 2009; Weber & Anderson, 2010). At both scales, nature is a positive influence on average LS.

The only variable that is consistently highly significant (with a negative coefficient) across all the scales is the standard deviation of LS. This is true in the samples “All” and “Unsatisfied.” This implies that as the inequality in LS increases amongst the population, the LS of individuals decreases. This is especially true amongst those that indicate that they have a low satisfaction with life (LS ≤ 5). In the case of the sample “Satisfied,” we found that only SA1 and SA2 were significant with positive coefficients. This shows that on a community level, those that have a high satisfaction with their lives (LS > 5), higher inequality increases their LS; that is, in the case of those that are already satisfied with their lives (LS > 5), seeing other individuals in their community with a lower LS serves to increase their own.

Problems with aggregation are also seen when we map average LS across Australia at the different spatial scales (Fig. 1). At the finest spatial scale, (SA1), the average LS scores range from 0 to 10, as shown on Fig. 1A. There are very few individuals in each SA1. However, as we aggregate to the state and territory scale, LS average scores show a much smaller range of variation. For example, at state and territory level the average ranges only from 7.87 to 7.99, as shown on Fig. 1E. Spatial aggregation brings the average scores much closer together, as the impact of outliers within the sample is reduced. This creates a situation where individuals reporting a LS score of 0–5 are averaged out. Those are the individuals that are most at risk within society, but are ignored when using regional or sub-national wellbeing indicators.

Any time data is downscaled or upscaled, as in this paper, assumptions are required about that data’s distribution. This inherently involves loss of data variation and an increase in uncertainty. The use of aggregated data that has greater uncertainty than certainty, may create spurious correlations, or correlation that cause incorrect inferences (Bordt, 2015). In this paper, not only is LS aggregated, but so too are the independent variables.

On the other hand, using large-scale data without aggregation creates big data problems. Looking at LS data of every individual in Australia is logistically unrealistic and provides a lot of “noise,” which makes it impossible to inform policies at reasonable scales.

### Comparing life satisfaction of the dissatisfied and the satisfied

Aggregating LS scores, and estimating the models separately, for satisfied and unsatisfied individuals shows how the relationship between objective variables and LS differs between the two groups (Table 4). Separating these individuals shows that information is being lost when aggregating measures for the entire population. We highlight some important examples below.

#### Environment

In our model, we used the NDVI as an indicator of natural capital, which has been shown to be a good proxy for ecosystem services and nature’s contributions to humans (Costanza et al., 1998; Bai et al., 2008; Taylor, Hahs & Hochuli, 2018).

The environment is often omitted in wellbeing indicators since it is hard to measure^3^3Selecting the most appropriate proxy for nature (from a very wide range of possibilities) is a non-trivial task, which we leave for future research. and is often not perceived to have a direct impact on LS. Previous studies have shown that proximity to nature has a direct impact on both physical and mental health (Takano, Nakamura & Watanabe, 2002; Mayer & Frantz, 2004; Howell et al., 2011; Donovan et al., 2013).

However, regressions “Unsatisfied” and “Satisfied” show that individuals with low LS (LS ≤ 5) perceive the environment differently than those with a high LS (LS >5). Those individuals with a high LS, we find NDVI significant at most scales (except at SA3 and SA4 levels) with a positive coefficient (*p* < 0.005). At the SA2 scale, environment has the third largest marginal value of all the variables. This shows that those satisfied with their lives, nature provides a positive contribution to their wellbeing at both small community scales and at the state level. The individuals have the time and the disposable income, and the health to enjoy nature.

However, for those individuals that have a relatively low LS, NDVI is not significant at any of the scales. One possible reason that NDVI is not significant in the “Unsatisfied” regression is that it only becomes important after basic subsistence requirements are met. While the average NDVI is similar at all scales between the two groups (Table S1), those with higher LS, on average, have higher incomes, work more hours, and have lower unemployment rates. They also have a lower proportion of individuals with long-term health conditions. The “Unsatisfied” sample may not have the health or time to properly enjoy nature, as the “Satisfied” sample.

#### Aggregation of income

Another major difference between regressions “Unsatisfied” (LS ≤ 5) and “Satisfied” (LS > 5) is in the variables of household income and poverty. For individuals with a lower LS, household income is significant and positive at only SA1 (*p* < 0.001). This implies that at larger scales, with aggregation, household income is not a major contributor to the reported low level of LS. Other factors have a great impact on LS, such as long-term health conditions. However, for individuals with a higher LS, household income is positively and highly significant at all scales besides state. For these individuals, additional household income does have a positive impact, which means that they may have more time and opportunities to use this additional household income.

The descriptive statistics in Table S1 shows that on average, individuals with relatively low LS have lower household disposable income ($60,240 in SA1s) compared to those with higher LS ($77,917 in SA1s). Thus, the stronger correlation between high LS and income may be due to a perception by individuals with higher LS that income is a major contributor to that higher LS, and perhaps these higher income levels are the social norm within their peer group of individuals satisfied with life.

The standard deviation of the household disposable income of the “Unsatisfied” sample is higher than that of the “Satisfied” sample, except at the SA1 level. This makes sense since a lower LS may not be solely caused by a lower household disposable income. Certain aggregated areas may have higher average household disposable incomes but lower levels of homeownership or higher levels of long-term health conditions, which decrease the average LS.

The “proportion of individuals below the poverty line,” is not significant at any level in the “Unsatisfied” sample, but is positive and significant at the SA3 (*p* < 0.001) and state/territory scales (*p* = 0.013) in the “Satisfied” sample. Research has shown that LS comes not from absolute income but relative income (Oscar, Gandy & Baron, 1998; Wilkinson & Pickett, 2006; Boyce, Brown & Moore, 2010; Oshio & Urakawa, 2014; Diermeier et al., 2017). Research has also indicated that the higher an individual’s income, the less satisfied they are with it (Ferrer-i-Carbonell, 2005). So their LS may increase when there is a larger range of incomes, including a larger proportion of individuals below the poverty line. At low-income levels, those relative effects are found to be smaller (McBride, 2001).

The lack of a strong correlation between lower LS and income may be explained by other factors contributing to a greater extent to LS. In regression 2, “Unsatisfied” sample, at the SA1 level the variables with the highest coefficients, or marginal contributions to LS, include physical activity, long-term health condition, and having at least a university degree (Table 4). These marginal effects are also the highest ones at the SA2 level.

#### Volunteer work

Volunteering is a proxy that has been used within previous research to represent social capital (Putnam, 2001). We find the hours spent doing volunteer/charity work is positively and highly significant in the “Satisfied” regression at SA1 through SA3 (*p* < 0.001) and not the “Unsatisfied” regression at any scale. This implies that individuals that are already satisfied with their lives, and have the time and resources to volunteer their time, receive a positive contribution from volunteering to their LS. Those that are not satisfied with their lives, find that volunteering neither provides a positive or negative contribution to their LS, probably because they have their own worries and do not have the time or other resources to enable them to volunteer.

#### Language

There are stark differences in the regression results for the “proportion of individuals that speak English well,” both across spatial scales and between samples (Table 4). It is worth noting that the proportion of individuals that speak English well is similar in both the “Unsatisfied” and “Satisfied” samples (Table S1). In this regard, the proportion of individuals who speak English well could indicate how individuals fit into their community. Fitting into a community is a critical aspect of wellbeing (Manderson, 2010). Having poor English skills while living in a community of English speakers can be isolating. This isolation is not only due to a difficulty in communication but can indicate a different set of values (Oishi et al., 2009; Graham & Markowitz, 2011). The lack of fluency in the local language may lead to lower health literacy, higher stress levels, decreased mental health, lower paying jobs, and other effects that overall decreasing LS (Aycan & Berry, 1996; Zanchetta & Poureslami, 2006). However, we acknowledge that this is not a complete measure of community cohesion. Local ethnic communities, speaking the same language, may provide some of that community cohesion (Yoon & Lee, 2010). A measure for this is not included in our model. At the SA4 and state scales, where the “proportion of individuals who speak English well” is significant and negative (*p* = 0.006 and *p* = 0.017, respectively), it is most likely capturing another demographic aspect.

### Future wellbeing indicators

Objective variables that are significant at the SA1 level often cease to be significant at the higher levels, especially at the state level. By focusing on only a single scale, information about objective factors that contribute toward LS may be lost through aggregation. For example, decisions regarding environmental policy based on state level indicators may fail to reflect the important contribution that the local environment, like community parks, makes to individual wellbeing at the local area level. As seen in regression 2 and 3 in Table 4 (“Unsatisfied” and “Satisfied”), the variables most significant to individuals with a lower LS differ from the variables most significant to individuals with a higher LS. By averaging the population over sub-national scales, individuals that are most vulnerable and most at risk within a population may be lost within the averages. Small areas that are excessively low (or high) in LS will be overlooked when averaging the entire population. Understanding the distribution and identifying regions where there is a dearth of critical contributors of LS, allows for targeted policies. Policies at the state, regional, and neighborhood scales take on different forms and produce different outcomes (Gabriel et al., 2010; Termeer, Dewulf & Van Lieshout, 2010; Gale, 2016).

When creating national or subnational wellbeing indicators, it is critical to understand the differences in the value sets that different portions of the population hold, arguing for focusing on finer and more appropriate scales. These value sets can vary due to a person’s age, where they grew up, whether they immigrated, and many other factors. For example, in Australia, the Indigenous population comprises 3.3% of the total population, just fewer than 800,000 individuals according to the 2016 census. However, the values they hold and the type and relative importance of factors that contribute to their LS has been found to differs greatly from others in Australia (Biddle, 2011; Yap & Yu, 2016). Even in countries without a large indigenous population, immigrants hold different value sets and recognize different contributors to their LS (Graham & Markowitz, 2011). This is also not restricted to cultural differences; males and females perceive contributors to wellbeing differently, as do the rich vs the poor, or old vs young. Aspects like safety, money, or family are perceived very differently in these different portions of the population.

Many wellbeing indicators are created based on information only from the sub-national or national scales. This creates a situation in which society is focusing on variables that may not be significant to individuals, and omitting those factors crucial at local level, such as the environment. Often, policies are set based on the assumption of improving variables contributing to human wellbeing. However, the scale of these policies is not often aligned with the most appropriate scale of the variable, potentially reducing the effectiveness of the policy intervention.

By only considering very fine scales of wellbeing, critical information around overall wellbeing of a nation may also be overlooked. Assessing wellbeing at a national scale illustrates the overall progress of the entire nation. A series of national level goals can provide a dashboard of wellbeing indicators; however, an aggregated overall indicator is also essential in measuring the wellbeing of the entire population (Costanza et al., 2016). A national level indicator is also important for comparing countries to determine which are moving toward or away from this goal.

## Conclusion

Although a range of different approaches to measuring a population’s wellbeing have been proposed over the last few decades, consensus has proved difficult to achieve. How a person perceives his or her wellbeing is difficult to assess, directly or indirectly. Nevertheless, measuring progress within society through improved individuals’ wellbeing requires a quantitative evaluation.

However, creating measures that assess every individual globally is impractical. Aggregated measures are commonly used to assess a population’s wellbeing. The process of aggregation, however, changes how we perceive and measure wellbeing, potentially making the measures inappropriate or meaningless. For example, does the average wellbeing of people within a region have a relationship with that regions average income? Or the average age of that population? It also changes the relationship between objective and subjective variables, where objective variables are meaningful at one scale and become meaningless at another. It is critical to understand how measuring wellbeing at different scales affects the measures themselves and what it means for the population.

This research has posed the question of what is the ideal scale for a wellbeing indicator? Unfortunately, there is no easy or simple answer to this question. It is critical to analyze the small scales to ensure that the individuals and communities that are most at risk are identified and assisted. Pinpointing the limiting variables of those communities provides critical information to addressing the dearth in self reported LS. This will also ensure that the inequality in LS diminishes within a region or country. On the other hand, sub-national and national indicators are crucial in assessing the progress and wellbeing of a nation as a whole.

Before a global consensus around a preferred indicator, or dashboard of indicators, is achieved, it is critical that the purpose of this indicator is defined. An indicator used for national comparisons will be vastly different to an indicator that tries to identify individuals most at risk within a population. Both of these indicators require different policy interventions and have varying impacts. For both types of indicators, a better understanding of the relationship between objective and subjective indicators (or perception and reality), the impact of aggregating at different special scales, aggregation methods, and data availability is required.

## Supplemental Information

10.7717/peerj.6502/supp-1 Supplemental Information 1 Average of each objective variable used in our regression and the standard deviation between the areas (reported in parentheses).

## Additional Information and Declarations

### Data Availability

The following information was supplied regarding data availability: The Household, Income and Labour Dynamics in Australia (HILDA) Survey (https://melbourneinstitute.unimelb.edu.au/hilda): RESTRICTED RELEASE 17 (Waves 1-16).
